# ZAD-Domain Is Essential for Nuclear Localization of Insulator Proteins in Drosophila melanogaster

**Published:** 2016

**Authors:** N.A. Zolotarev, O.G. Maksimenko, P.G. Georgiev, A.N. Bonchuk

**Affiliations:** Institute of Gene Biology, Russian Academy of Sciences, Vavilova str. 34/5, Moscow, 119334, Russia

**Keywords:** insulators, chromatin, transcription factors

## Abstract

Many arthropod zinc-finger transcription factors contain a N-terminal domain called ZAD (Zinc-finger Associated Domain), which consists of four cysteines coordinating a single zinc ion. Dimerization ability has been shown for several ZAD-domains. The functional role of this domain is poorly understood. In this paper, we demonstrate that a point mutation within the ZAD-domain of the Zw5 insulator protein disrupts its nuclear localization without affecting its dimerization ability. The importance of the ZAD-domain for nuclear localization has also been shown for the Pita and Grauzone proteins. Therefore, one of the ZAD-domain functions is control of the nuclear localization of transcription factors.

## INTRODUCTION

**Fig. 1:**
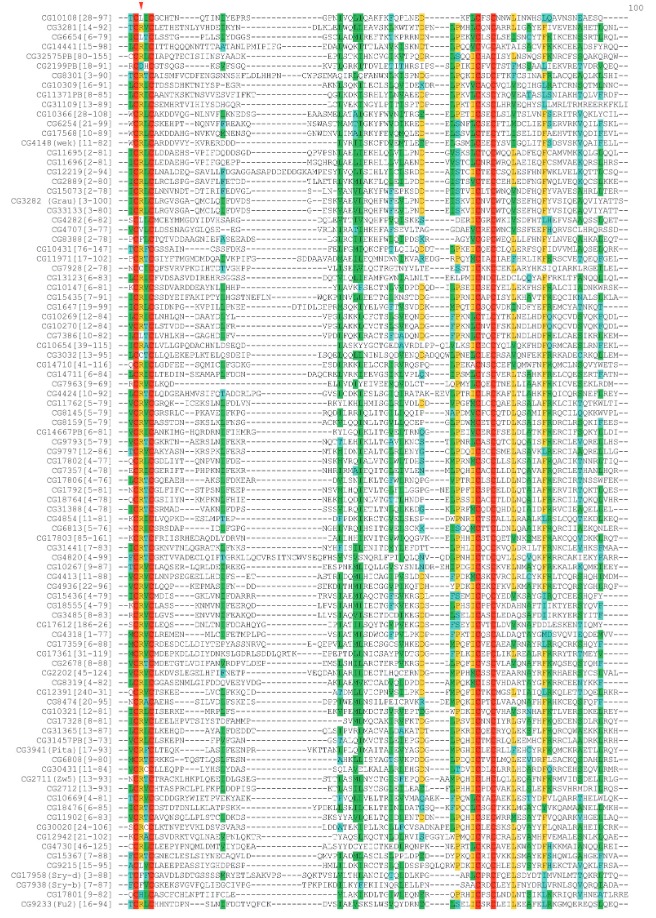
Multiple sequence alignment of *Drosophila* ZAD-domain amino-acid sequences. Red arrow indicates the position of conserved arginine.

**Fig. 2:**
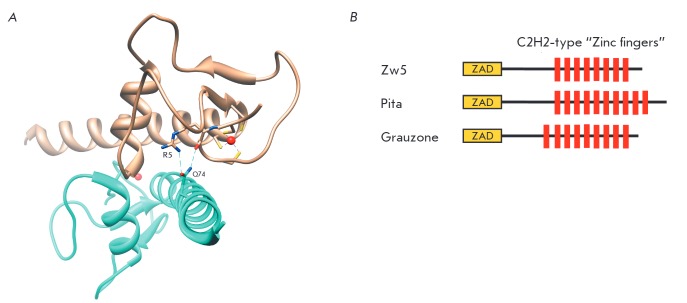
A – Crystal structure of ZAD-domain dimer from Grau protein (1PZW in PDB). Hydrogen bonds between R5 (residue homological to R14 of Zeste-white 5 protein) and Q74 are shown; also shown are four cysteines coordinating the zinc-ion. B – Domain structure of the Zeste-white 5, Pita, and Grauzone proteins.

Proteins with a “zinc fingers” DNA-binding domain of C2H2-type are the largest class of transcription factors in higher eukaryotes [1]. The C2H2-domains usually form clusters, some of which are responsible for highly specific binding to DNA. A subclass of C2H2-type “zinc fingers” transcription factors was found in arthropods: its representatives contain a specific domain at the Nterminus, called ZAD (Zinc-finger Associated Domain), which contains four cysteines coordinating a single zinc ion [2, 3]. *Drosophila melanogaster *cells contain over 90 transcription factors (*Fig. 1*) with C2H2- and ZAD-domains [3]. The genomes of other arthropods may encode from four (*Daphnia pulex*) to 120 (anopheles mosquito) factors of this class. Determination of the crystal structure of the ZAD-domain from the Grauzone (Grau) factor revealed that it is a dimer (*Fig. 2A*) [4]. In addition, it has been demonstrated *in vitro *that the ZADdomains of the Serendipity-δ and Weckle proteins also form dimers [5, 6]. To date, this family of transcription factors remains almost unexplored, with functional roles established only for a few of them [7-9]. Three transcription factors with ZAD-domains (Pita, ZIPIC and Zw5) can be classified as insulator proteins (*Fig. 2B*) [10, 11]. Insulators are regulatory elements that block interaction between an enhancer and a promoter only if they are located between them [12, 13]. Recently, it has been shown that insulators may be involved in establishing distal interactions and the organization of chromosome architecture [14, 15]. The Zw5 protein was found in the SCS insulator, located on the boundary of a heat shock 70 gene cluster [10]. Pita and ZIPIC proteins were initially identified as partners of the insulator CP190 protein, which is believed to play the key role in the formation of chromatin architecture [11]. All insulator proteins bind to specific nucleotide sequences, 9–15 bp in length, whose multiplication creates an effective insulator [10, 11, 16]. A genome-wide analysis showed that Zw5, Pita, and ZIPIC preferentially bind to gene promoters [11]. It is assumed that the ZADdomain may be involved in the organization of distal interactions between the remote binding sites of one insulator protein [15].

In the present study, we investigated a point mutation in the ZAD-domain of the Zw5 protein, which leads to a lethal phenotype. It has been shown that this mutation disrupts nuclear localization of the Zw5 protein. The ZAD-domains of the other two proteins, Pita and Grau, are also essential for nuclear localization of these proteins. Therefore, one of the functions of the ZAD-domain is the regulation of the nuclear localization of transcription factors.

## MATERIALS AND METHODS

**Expression and purification of the proteins**

The DNA encoding ZAD-domains of the Zw5 protein (wild-type and R14G mutant) of *Drosophila *was cloned into the pET32a(+) plasmid in a frame with six histidine residues and Thioredoxin. The plasmids were used to transform competent cells of the *Escherichia coli *strain BL21(DE3). Protein expression was induced with 1 mM IPTG. 0.2 mM ZnCl2 was added prior to the induction, and the culture was incubated overnight at 18 °C on a shaker. The bacterial cells were destroyed by sonication in 50 mM HEPES-KOH buffer (pH 7.6), containing 500 mM NaCl, 20 mM imidazole, 5 mM β-mercaptoethanol, and 1 mM PMSF supplemented with 1:500 Proteinase inhibitor cocktail VII (Calbiochem). The proteins were isolated from the bacterial cell lysate by affinity chromatography on Co-IDASepharose. The elution was performed using 50 mM HEPES-KOH buffer (pH 7.6), containing 500 mM NaCl, 250 mM imidazole, 5 mM β-mercaptoethanol.

**Chemical cross-linking of the proteins**

Following the isolation, the proteins were dialyzed against PBS buffer. Glutaraldehyde was added to the preparations to a final concentration of 0.01 or 0.1%, and they were incubated at room temperature for 10–15 min. The reaction was quenched by adding glycine to a concentration of 50 mM and incubated at room temperature for 15 min. The products of the cross-linking reaction were visualized by electrophoresis in polyacrylamide gel, followed by silver staining.

**Image staining of the S2 cells of *D. melanogaster***

cDNAs encoding either full-length wild-type proteins or mutant ones were cloned into a plasmid in frame with 3×FLAG-peptide for transient expression under the control of the actin 5C gene promoter. The cells were transfected according to the standard procedure using the Cellfectin reagent (Invitrogen). The staining was performed on the third day after the transfection.

1 ml of the suspension of S2 cells was applied to a cover glass of a 35-mm Petri dish. It was incubated overnight at 24 °C to precipitate the cells on the glass. The medium with non- adhered cells was removed.

All solutions were applied to the wall of the dish, so as not to wash off the cells from the glass. The incubation was performed at room temperature on a shaker at low speed.

The glass was washed with 1 ml of phosphate-buffered saline (PBS, 2 × 5 min). The cells were fixed with 1 ml of the fixation solution (2% formaldehyde, 50 mM MgCl_2_ in PBS) for 20 min. They were washed with 1 ml of PBS (3 × 5 min). To increase the permeability of the cell membranes, the cells were treated with 1% Triton X-100 in PBS for 10 min. The cells were washed with PBS (1 ml, 3 × 5 min). They were incubated in a blocking solution (1% bovine serum albumin (BSA), 0.05% Tween-20 in PBS, 2 × 30 min) and incubated with 1 ml of the blocking solution with primary antibodies (1:30 anti-lamin (from the collection of the University of Iowa), 1:300 anti-FLAG (Sigma)). Then they were washed with the solution of 0.25% BSA, 0.05% Tween-20 in PBS (3 × 15 min), incubated with 1 ml of the blocking solution with secondary antibodies conjugated with a fluorophore (Invitrogen) and TO-PRO-3 Iodide for 1 h. They were washed with the solution of 0.25% BSA, 0.05% Tween-20 in PBS (3 × 15 min), and washed with 1 ml of PBS for 5 min. The glass was then washed with water, and the excess water was removed. A Vectashield medium was added for fixing on the cover glass. The edges of the cover glass were, coated with varnish.

## RESULTS

**Lethal mutation in the ZAD-domain of Zw5 protein does not affect its ability to dimerize**

*The dwg *(*deformed wings*) gene that encodes the Zw5 protein is expressed primarily during embryogenesis. There are many mutations described for the *dwg *gene that produce a lethal phenotype, suggesting that Zw5 plays an important role in embryonic development. One of well-characterized mutations, *dwg^8^*or *zw5^62jl^*, leads to substitution of arginine for glycine at position 14 (R14G) of the ZAD-domain [10]. This recessive mutation is lethal at the larval stage. To prove that the *zw5^62jl^*mutation is the one responsible for the lethal phenotype, a construct was obtained in which the *dwg *gene was under the control of the *hsp83 *gene promoter. *White *gene that determines eye pigmentation was used as the reporter gene to identify the transformants. Four transgenic lines with a single insertion of the construct were obtained as a result of the transformation into *Drosophila *embryos. All transgenes complemented the lethal phenotype of the* zw562jl *mutation, confirming the role of this mutation in the manifestation of the lethal phenotype.

Therefore, a point mutation in the ZAD-domain leads to a complete functional inactivation of Zw5. Based on this data, it can be concluded that the presence of R14 in the ZAD-domain is essential. The alignment of the ZAD-domain sequences from different proteins demonstrated that most ZAD-domains contain an arginine residue at position 14 (*Fig. 1*). Out of 93 transcription factors with a ZAD-domain in the *Drosophila* genome, 81 have arginine at position 14 (R14). In five proteins, arginine is replaced with leucine; in three, with phenylalanine; in two, with cysteine; and in one, with aspartate and histidine. Previously, it has been suggested that R14 participates in the ZADdomain dimerization [4], since it (R5 in Grau protein) is involved in the formation of hydrogen bonds with Q74 (*Fig. 2A*), although these residues are only a small part of an extended dimerization interface between two ZAD-domains. In order to test this hypothesis, cDNAs encoding the ZAD^wt^ and ZAD^R14G^ domains fused to Thioredoxin were cloned into a pET32a(+) vector and expressed in *E. coli *cells. It was found that the mutant ZAD-domain is expressed in much lower quantities than the wild-type domain. This may indicate a lesser stability and disruption of the proper conformation of the mutant ZAD-domain expressed in bacteria.

**Fig. 3:**
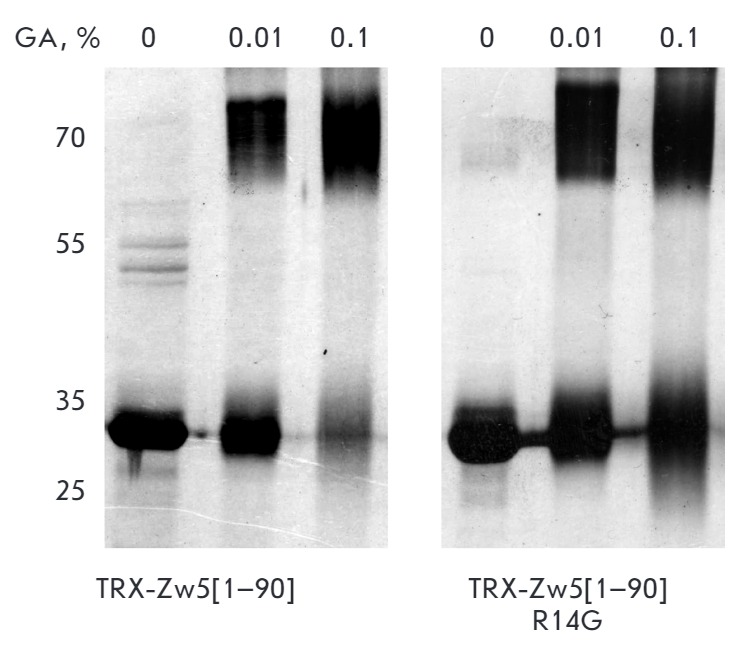
Results of chemical cross-linking with glutaraldehyde (GA) of the wild-type Zw5 ZAD-domain and the domain with R14G mutation, fused with Thioredoxine (TRX). Protein concentration is 10 μM.

To compare the dimerization of the normal and mutant ZAD-domains, we cross-linked the ZAD-domains using glutaraldehyde. As can be seen from *Fig. 3*, at a concentration of 10 μM both ZAD-domains form dimers with a similar efficiency. Therefore, the R14G mutation does not affect the ability of the Zw5 ZADdomain to dimerize *in vitro*, but we cannot exclude the possibility of reduced stability of the dimer at low protein concentrations in the cell.

**ZAD-domains are essential for nuclear localization of the proteins in Drosophila S2 cells**

**Fig. 4:**
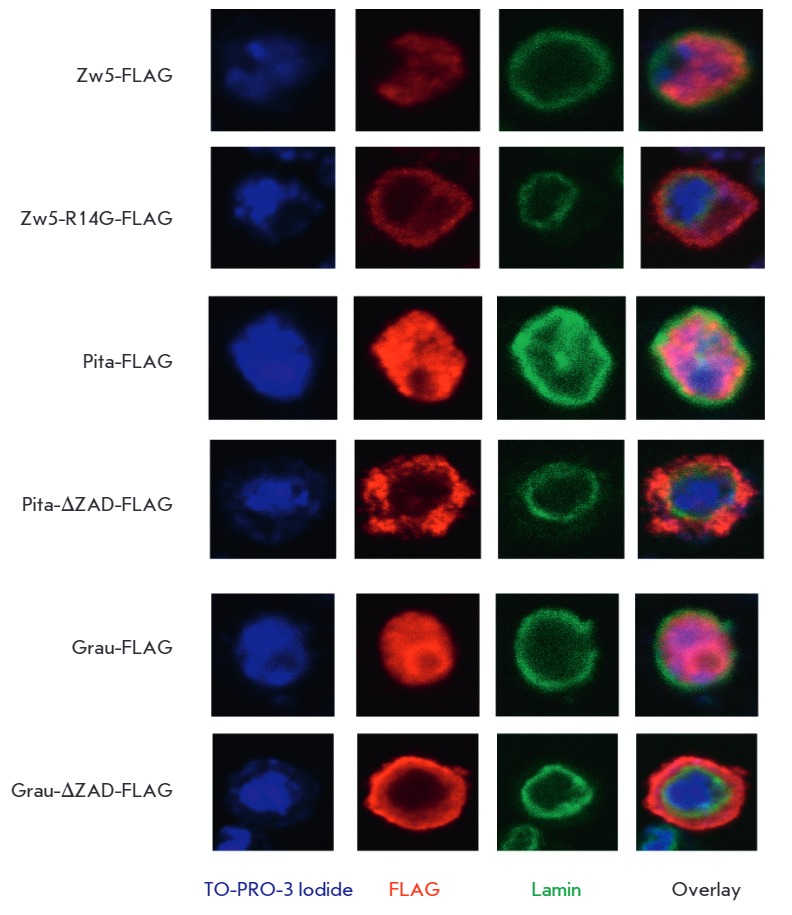
Immunocytochemical staining of S2-cells expressing proteins with a disrupted ZAD-domain and wild-type proteins fused with the 3xFLAG-peptide.

It was found that the mutant ZAD-domain retains its ability to dimerize; therefore, the next task was to study the distribution of the normal and mutant Zw5 proteins in *Drosophila *S2 cells. For this purpose, we had created expression vectors in which the genes encoding the normal and mutant Zw5 proteins fused to 3×FLAG epitope were under the control of the actin promoter. These vectors were used to transfect S2 cells, and the protein distribution was determined using antibodies to 3×FLAG epitope and lamin, and TO-PRO-3 Iodide dye, which stains DNA (*Fig. 4*). The Zw5-3×FLAG protein is localized mainly in the nucleus. Surprisingly, the Zw5R14G-3×FLAG protein was found almost exclusively in the cytoplasm. Thus, the point mutation disrupts nuclear localization of the Zw5 protein, which explains the lethal effect of the *zw5^62jl^*allele. According to the data of the NucPred service [17], the Zw5 protein does not contain nuclear localization signals.

To elucidate the role of the ZAD-domain in the nuclear localization of the proteins, we examined two other well studied proteins with the ZAD-domain: Pita and Grau [4, 8, 11]. These proteins have the same structure, similar to that of Zw5: the ZAD-domain is located at the N-terminus and a group of C2H2 type “zincfinger” domains forming a cluster at the C-terminus (*Fig. 2B*). It has been shown that “zinc finger” domains may define the nuclear localization of the proteins [18, 19]. According to the data of the NucPred software [17], Pita has no nuclear localization signals (NLS) and Grauzone has NLS in its central part (170–190 amino acids). The PredictProtein service [20] predicts nuclear localization for all three proteins with a low degree of significance for Zw5 and Pita and with a high degree for Grauzone.

cDNAs, encoding the Grau and Pita proteins with and without the ZAD-domain, were fused with 3×FLAG epitope and incorporated into an expression vector under the control of the actin promoter (Act5C). The resulting constructs were used to transfect S2 cells, and the distribution of the protein in the cells was studied using antibodies to 3×FLAG epitope and lamin (*Fig. 4*). The Pita-3×FLAG and Grau-3×FLAG proteins were located predominantly in the nucleus. Staining of the endogenous Grau, Zw5, and Pita proteins (data not shown) revealed their uniform nuclear-cytoplasmic distribution. Deletion of the ZAD-domain led to localization of the FLAG-antibodies predominantly in the cytoplasm, which can be attributed to a loss of the ability to enter the cell nucleus by proteins without a ZADdomain (*Fig. 4*).

## DISCUSSION

Our results suggest that point substitution of arginine for glycine at position 14 (R14G) of the ZAD-domain disrupts the nuclear localization of the Zw5 protein. R14 in the ZAD-domain of Zw5 corresponds to the fifth amino acid residue of the ZAD-domain of the Grau protein. According to its crystal structure, the side chain of this residue is exposed at the surface and is capable of forming a hydrogen bond with Q74 [4]. Based on these data, the authors [4] suggested that arginine-5 is involved in the dimerization of the Grau ZAD-domain. However, according to our results, arginine-14 in the ZAD-domain of Zw5 is not essential for protein dimerization. It is possible that the structure of different ZAD-domains can vary significantly, which explains the preferable homodimerizaiton of ZAD-domains (unpublished data).

The putative localization of arginine-14 on the surface of the ZAD-domain may explain the role of this residue in the interaction with the proteins involved in nuclear import. The ZAD-domains of the Pita and Grau proteins have sequence similarity with each other and with the ZAD-domain of Zw5 of less than 58%. However, these ZAD-domains are also required for nuclear localization of the respective proteins.

Interestingly, bioinformatic approaches predicted nuclear localization signals in the central part of Grau and an absence of such signals in the Pita and Zw5 proteins. However, the Pita protein is present in cells as two isoforms which differ by the presence of the first 60 amino acids of the ZAD-domain [according to Flybase]. According to experimental data, subcellular localization of transcription factors with a ZAD-domain is the subject of a complex regulation. For example, it has previously been demonstrated that the nuclearcytoplasmic localization of the Weckle protein changes during development [6]. The ZAD-containing Trade Embargo protein, which binds to chromatin, is evenly distributed between the nucleus and the cytoplasm [21].

## CONCLUSION

Our results show that nucleocytoplasmic distribution of ZAD-containing transcription factors appears to have regulatory importance and that the ZAD-domains are essential for this process.

## Abbreviations

ZAD: Zinc-finger Associated Domain
IPTG: Isopropyl-β-D-thiogalactopyranoside
PMSF: phenylmethylsulfonylfluoride
